# Preparation and Characterization of Blended Films from Quaternized Hemicelluloses and Carboxymethyl Cellulose

**DOI:** 10.3390/ma9010004

**Published:** 2015-12-23

**Authors:** Xian-Ming Qi, Shi-Yun Liu, Fang-Bing Chu, Shuai Pang, Yan-Ru Liang, Ying Guan, Feng Peng, Run-Cang Sun

**Affiliations:** 1 Beijing Key Laboratory of Lignocellulosic Chemistry, Beijing Forestry University, Beijing 100083, China; qxmfly@foxmail.com (X.-M.Q.); Liushiyun_Daisy@163.com (S.-Y.L.); chufangbingcfb@163.com (F.-B.C.); pangshuai2012@126.com (S.P.); l8141586@163.com (Y.-R.L.); rcsun3@bjfu.edu.cn (R.-C.S.); 2 School of Forestry and Landscape Architecture, Anhui Agricultural University, Hefei 230036, China; xiaomi1231@163.com

**Keywords:** blend film, quaternized hemicelluloses, carboxymethyl cellulose

## Abstract

Utilization of hemicelluloses from biomass energy is an important approach to explore renewable resources. A convenient, quick, and inexpensive method for the preparation of blended films from quaternized hemicelluloses (QH) and carboxymethyl cellulose (CMC) was introduced into this study. QH and CMC solution were first mixed to form homogeneous suspension, and then were dried under vacuum to fabricate the blended films. The FT-IR and XRD results indicated that the linkage between QH and CMC was due to the hydrogen bonding and electrostatic interaction. From the results of mechanical properties and water vapor permeability (WVP), the tensile strength of the blended films increased with the QH/CMC content ratio increasing in appropriate range, and the WVP of the blended films decreased. The maximum value of tensile strength of blend film achieved was 27.4 MPa. In addition, the transmittances of the blended films increased with the decreasing of QH/CMC content ratio. When the weight ratio (QH: CMC) was 1:1.5, the blend film showed the best light transmittance (45%). All the results suggested that the blended films could be used in areas of application in the coating and packaging fields from the good tensile strength, transmittance, and low WVP.

## 1. Introduction

With the decrease in fossil fuels, much attention has been paid to investigate the new biodegradable materials. The increased social awareness for sustainable development gather momentum in favor of materials from renewable resources [1,2,3]. Therefore, polysaccharides have become the main part of natural-based materials which are particularly advantageous due to their biocompatibility, biodegradability, and non-toxicity [4,5,6]. Production of environmentally friendly packaging materials based on polysaccharides from renewable resources are also expected to substitute for petroleum-based packaging materials [7]. Cellulose and hemicelluloses are the most and the second most abundant polysaccharides in biomass, respectively [8,9,10,11]. Some studies have been widely studied for preparing films from them. Biocomposite films based on quaternized hemicelluloses and montmorillonite showed good thermal properties by the addition of clay nanoplatelets [12]. Bacterial cellulose as reinforcement was added in the arabinoxylan films, it could effectively increase the stiffness and strength of the films [13]. In addition, hydrophobic films formed by acetylated bleached hemicelluloses and acetylated cellulose showed the improvement of thermal and mechanical properties [14]. Therefore, the films based on hemicelluloses and cellulose have a prospective application in many fields.

Various derivatives of cellulose have been synthesized and used in practical applications. Carboxymethyl cellulose (CMC) is one of the most widely applied cellulose derivatives and it has good film forming property, which can form transparent films and possess high mechanical strength [15,16]. It contains a hydrophobic polysaccharide backbone and many hydrophilic carboxyl groups, showing amphiphilic characteristic [17]. CMC is an acidic polysaccharide, due to its a number of carboxylic substituents [18]. In addition, hemicelluloses are also the excellent materials for the film preparation [19,20,21]. Quaternized hemicelluloses (QH) are derivative of hemicelluloses which have highly solubility and cationic property because of the cationic agents and hydroxide radical in QH [22].

Blending is a main method for fabricating films by mixing two components, which is a simple and effective method to improve the properties of films [23,24]. When the two components are compatible, more homogeneous structure and better physicochemical properties of the blended films can be obtained than that of films from individual components [25]. In this article, quaternized hemicelluloses (QH) were obtained from bamboo hemicelluloses by esterified with 2,3-epoxypropyltrimethyl ammonium chloride [26]. Then, QH and CMC as the positive and negative charged polyelectrolyte, respectively, could form the polyelectrolyte complex through intermolecular hydrogen bonding and strong electrostatic attraction. A series of novel blended films that are non-toxic, renewable, and biodegradable were prepared from the two kinds of biopolymer according to a predetermined ratio. The structural analyses of the blended films were determined by FT-IR and XRD. The morphological structure was demonstrated by scanning electron microscopy (SEM) and atomic force microscopy (AFM). The mechanical properties and light transmittance were studied by tensile test and UV/Vis spectrophotometer. The water vapor permeability (WVP) of the blended films was performed according to the standard ASTM E 96/E 96M-05 [27]. The relationship between the structure and their physicochemical properties of the blended films was also discussed in this study.

## 2. Experimental Section

### 2.1. Materials

The hemicelluloses was extracted and purified from the *phyllostachys pubescens* (Meishan, Sichuan Province, China). The sugar composition of hemicelluloses was 83.6% xylose, 5.1% arabinose, 4.2% glucose, 0.4% galactose, and 6.8% glucuronic acid (relatively molar percent). The molecular weight obtained by gel permeation chromatography (GPC) showed that the native hemicelluloses had an average molecular weight (Mw) of 13,420 g·mol^−1^ and a polydispersity of 4.1, corresponding to a degree of polymerization of 88. Sodium hydroxide was purchased from Beijing Chemical Works (Beijing, China). 2,3-epoxypropyltrimethyl ammonium chloride (ETA) was obtained from Sigma-Aldrich Co., Ltd. (St. Louis, MO, USA) CMC, the degree of substitution is 0.78, was supplied by Tianjin Jinke Fine Chemical Research Institute (Tianjin, China). The weighed average molecular weight of CMC is 1.9 × 10^4^, and the viscosity is 800–1200 Pa·s. All reagents mentioned above were directly used without further purification.

### 2.2. Preparation of Quaternized Hemicelluloses

The synthesis of QH was carried out in a three-necked flask fitted with a mechanical stirrer and a reflux condenser. 0.66 g of dried hemicelluloses were dissolved in 5 mL distilled water at 60 °C for 30 min. An aqueous solution of sodium hydroxide (the molar ratio of NaOH to ETA, 0.75) was added, followed by adding of ETA (the molar ratio of ETA to anhydroxylose units in hemicelluloses, 2.0). The mixture was stirred at 60 °C, for a total period of 5 h. Upon completion of the reaction, the mixture was cooled to ambient temperature. Then, the reaction mixture was filtered off and thoroughly washed with ethanol, and dried in a vacuum oven at 45 °C for 24 h.

### 2.3. Preparation of Blended Films

QH were dissolved in distilled water and stirred for 6 h. The suspension was centrifuged at 4000 rpm for 10 min, and the supernatant was stored in refrigerator at 4 °C for the use of film preparation. The insoluble solids were dried to calculate concentration of the supernatant. CMC was dissolved in distilled water at 60 °C for 30 min under magnetic stirring to prepare the solution with concentration of 2 wt %. The QH and CMC solution concentrations were all 2 wt %, and they were mixed under magnetic stirring energetically at 60 °C. The mixing solution was degassed by rotary evaporator at room temperature for 2 h, after which per 10 mL solution was cast into a 5.5 cm diameter plastic petri dishes and dried under vacuum at 40 °C for 36 h. Then the blended films were obtained and peeled off carefully. The different volume ratios of QH to CMC are shown in Table 1.

**Table 1:** Composition of the blended films.

Sample Code	QH: CMC (*V*/*V*)
Film 1	1.0:1.0
Film 2	1.0:1.5
Film 3	2.0:1.0
Film 4	1.5:1.0

### 2.4. FT-IR Spectroscopy

FT-IR spectra of QH, CMC, and QH/CMC blended films were recorded using a Nicolet iN 10 Fourier transform infrared spectrometer (Thermo Nicolet Corporation, Madison, WI, USA). QH and CMC powder were prepared by grinding with potassium bromide and laminating. FT-IR spectra were recorded in the spectra range from 4000–650 cm^−1^. The blended film samples were measured under the ATR mode with spectral range of 4000–650 cm^−1^.

### 2.5. X-ray Diffraction

X-ray diffraction patterns of QH, CMC, and QH/CMC blended films were recorded with a XRD-6000 X-ray diffractometer (Shimadzu, Kyoto, Japan), using Nickel-filtered Cu Kα radiation (λ = 0.154 nm) at 40 kV and 30 mA in the 2θ range of 5°–45° at a speed of 5°·min^−1^.

### 2.6. Atomic Force Microscopy (AFM)

Surface roughness of the films was measured using a Multimode 8 (Bruker, Billerica, MA, USA), Atomic force microscope (AFM), operated in tapping mode. Silicon cantilever was used throughout the study with the nominal tip radius of 10 nm curvature. All scans were performed in air, at room temperature. Height and phase contrast images were recorded simultaneously with a resonance frequency between 250 and 300 kHz and a scan angle of 0°.

### 2.7. Scanning Electron Microscopy (SEM)

Scanning electron micrographs of the films, brittle fractured in liquid nitrogen, were taken with S-3400N scanning electron microscope (Hitachi, Tokyo, Japan). Prior to the SEM analysis, the films were sputter coated with a thin layer of gold. The acceleration voltage was set to 15 kV and magnification was 1500×.

### 2.8. Light Transmittance

The light transmittance of the blended films were performed by a UV-Vis spectrophotometer (UV 2300, Tech Comp, Shanghai, China) at wavelength of 200–800 nm. The films strips were attached to the surface of the cuvette with the size of 12 mm × 60 mm, taking cuvette as a reference.

### 2.9. Tensile Properties

Tensile strength and breaking elongation of films were measured using a miniature universal testing machine (CMT6503, SANS Technology stock Co., Ltd., Shenzhen, China), with a load cell of 100 N, operating in tension mode. The initial grip distance was 20 mm, and the rate of grip separation was 5 mm/min. The specimens were cut to the size of 30 mm × 10 mm. At least three repeated measurements of each film were tested and the average value was used to determine mechanical properties.

### 2.10. Water Vapor Permeability

The standard ASTM E 96/E 96M-05 was used to determine water vapor permeability (WVP) [27]. Aluminum cups were filled with 15 g anhydrous calcium chloride as desiccant and the film samples were mounted over the cups at room temperature. The desiccant was dried at 240 °C for 3–5 h before use. Aluminum cups were then placed in a cabinet containing water. WVP was determined by

$$\text{WVP}=\frac{qd}{t\text{s}\Delta P}$$

where *q*/*t*, *d*, s, Δ*P* are the slope of the weight increase *versus* time (g/s), the film thickness (cm), the film area exposed to moisture (4.91 cm^2^), and the difference of the vapor pressure between the two sides of films (23.76 mmHg), respectively.

## 3. Results and Discussion

### 3.1. FT-IR Analysis

**Figure 1:**
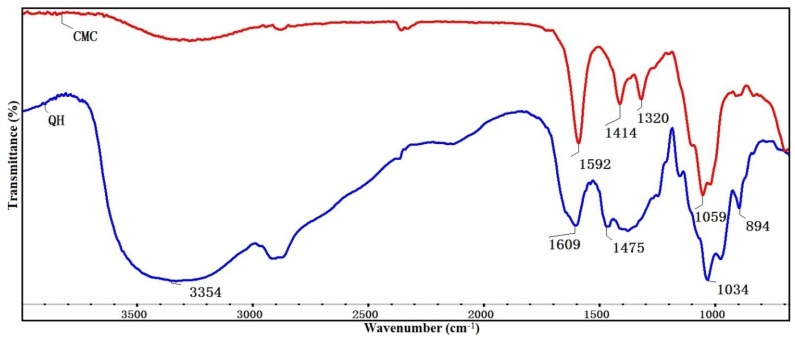
The FT-IR spectra of CMC and QH.

**Figure 2:**
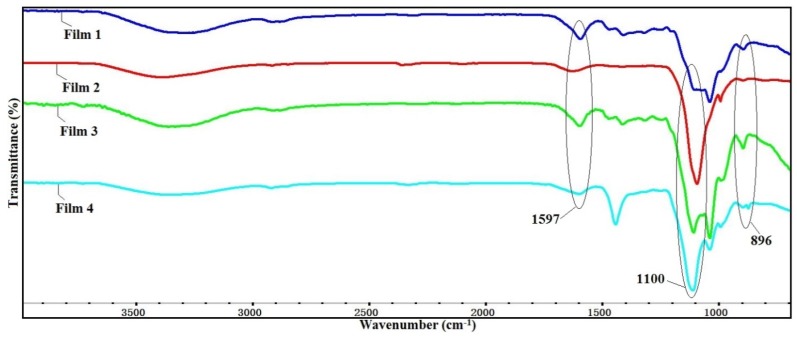
The FT-IR spectra of four blended films.

Figure 1 shows the FT-IR spectra of CMC and QH. As can be seen, the absorption band at 1592 cm^−1^ is assigned to asymmetrical COO– stretching of CMC [28]. The signals at 1414 and 1320 cm^−1^ are related to the symmetrical stretching vibrations of the carboxylate groups and C–H bending, respectively [29]. The broad peak at 1059 cm^−1^ is attributed to the stretching of C–O–C [30]. In the spectrum of QH, the absorption band at 3354 cm^−1^ corresponds to –OH stretching vibration, and the vibration at 1609 cm^−1^ reflects the blending mode of absorbed water [31]. The stretching vibration at 1034 cm^−1^ and 894 cm^−1^ which can be ascribed the C–O–C and the β-glycosidic bond, respectively [32]. In addition, a new absorption peak at 1475 cm^−1^ appeared in QH, which originates from the bending vibration of –CH_3_ and –CH_2_ in quaternary ammonium group. It suggests that the hemicelluloses were modified successfully [33]. Compared with the FT-IR spectra of pure CMC and QH, the spectra of blended films are shown in Figure 2. The stretching vibration of the C–O–C was broadened and shifted evidently to the 1100 cm^−1^, which was caused by the overlap of the ether bond in both CMC and QH. The absorption peaks at 1597 cm^−1^ and 896 cm^−1^ of blended films are attributed to asymmetrical COO– stretching and β-glycosidic bond, respectively. The broad absorption band at 3300–3500 cm^−1^ is also assigned to –OH stretching of hydrogen bonds. The blended films were fabricated by mixing the QH with CMC, abundant hydrogen bonds were therefore formed between the QH and CMC chains. Furthermore, plenty of ammonium cations are on the surface of QH, and there are many carboxyl anions in CMC chains. This resulted in the electrostatic interactions that were formed between the QH and CMC. Therefore, the blending of QH and CMC was mainly due to the hydrogen bonds and electrostatic interactions between them.

### 3.2. X-ray Analysis

**Figure 3:**
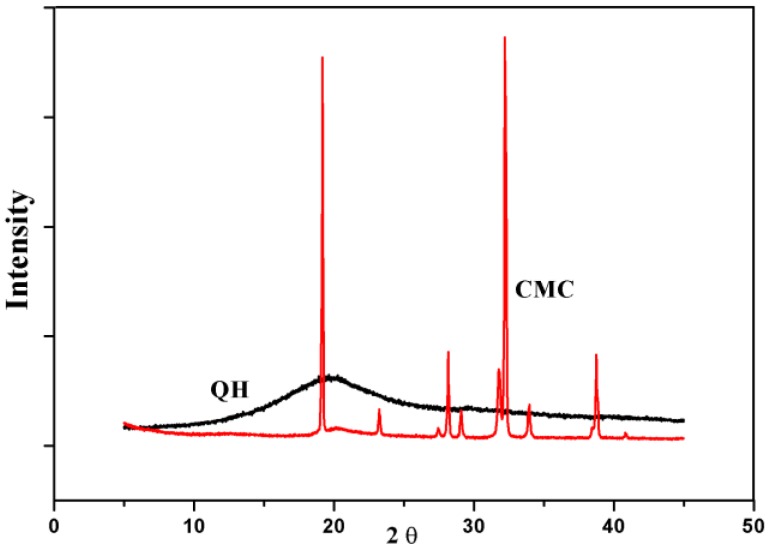
The XRD patterns of QH and CMC.

**Figure 4:**
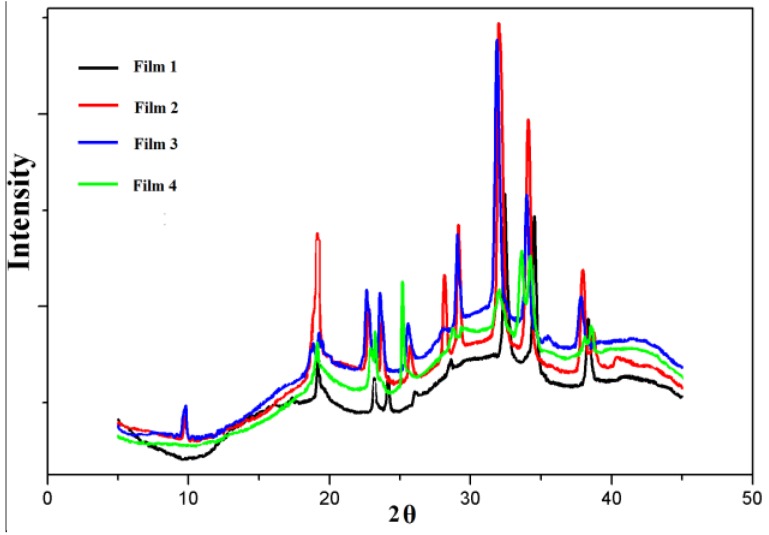
The XRD patterns of Film 1, Film 2, Film 3, and Film 4.

The impact of chemical modification on the crystal structure was further evaluated by using X-ray diffraction analysis. The X-ray diffraction patterns of QH and CMC are shown in Figure 3. The diffraction peak of QH is at 2θ = 19.9° [34]. It was found that the diffraction peak intensity of QH was lower than that of CMC, which was due to the amorphism of hemicelluloses. The diffraction of CMC consists of two major crystalline peaks are at 2θ = 18.9° and 32.6°, respectively, which are the characteristic of cellulose I [35,36]. Moreover, the intensity of CMC diffraction peak at 2θ = 28.2° and 38.7° become weak, because the hydroxyl in cellulose are substituted by carboxyl groups. It could be partially explained by molecular rearrangement of CMC. The XRD patterns of the blended films showed that the major diffraction peaks are at 2θ = 19.0°, 33.0°, 34.2°, and 37.8° in Figure 4. It suggested that the diffraction peaks of composite films were overlapped and shifted. The degree of crystallinities had the following order: CMC > Film 4 > Film 3 > Film 2 > Film 1 > QH, and the crystallinity values of them were present in Table 2. Theoretically, the crystallinity value of the blended films should increase with the increase of CMC amount, because CMC is crystalline and QH is amorphous. However, the XRD results of blended films indicate that the crystallinity value of the blended films do not show the obvious positive correlation with CMC amount. The intermolecular hydrogen bonding and electrostatic interaction are the driving force of blended films between QH and CMC, which may change the crystal structure of the blended films. The intensity of the driving force is different with the increase of QH/CMC content. Moreover, the crystalline region of CMC and the amorphous region of QH also have influence on the final degree of crystallinity of the blended films. Therefore, the crystallinity values of the blended films do not show obvious regular change with the QH/CMC content ratio.

**Table 2:** The degree of crystallinity of QH, CMC, and the blended films.

Sample Code	QH	CMC	Film 1	Film 2	Film 3	Film 4
Crystallinity(%)	9	84.6	27.7	33.4	34	35.9

### 3.3. Morphology of Blended films

**Figure 5:**
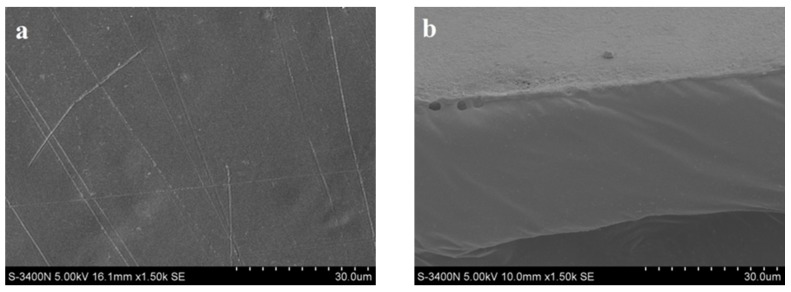
SEM images of the surface (**a**) and cross section (**b**) of Film 4.

The SEM images of the surface and cross section of the blend film are present in Figure 5. As can be seen from Figure 5a, the surface of the film is smooth and homogeneous without any aggregation. It suggested that the QH and CMC were diffused evenly in the film. In the microstructure of the film cross section in Figure 5b, a dense and smooth structure of blended film suggested that strong interaction force existed between QH and CMC. QH and CMC have a positive charge and negative charge, respectively. In addition, abundant of hydroxyl groups were existed in the surface of QH and CMC. Therefore, hydrogen bonding and electrostatic attraction between the oppositely charged polymers are the driving force for the formation of tight and smooth films.

**Figure 6:**
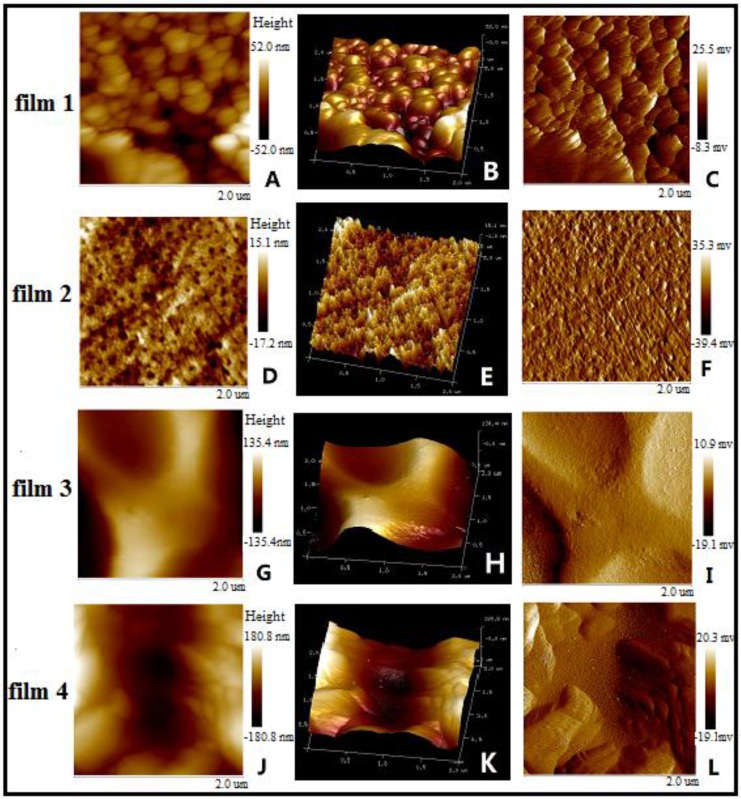
AFM height images (**A**,**D**,**G**,**J**), 3D topography (**B**,**E**,**H**,**K**), and phase topography (**C**,**F**,**I**,**L**) of the blend Film 1, Film 2, Film 3, and Film 4.

The surface nanotopography and toughness of the four blended films were investigated by AFM. The height images, 3D topography, and phase topography of the films are shown in Figure 6. From the height images and 3D topography of films, it was obviously found that the surfaces of blended Film 1 and Film 2 were more flat than those of blended Film 3 and Film 4. The roughness values were calculated in the areas of 2 μm × 2 μm, and the roughnesses of Film 1, Film 2, Film 3, and Film 4 were 11.4, 3.48, 40.7, and 57.3 nm, respectively. The roughnesses of the blended films increased with the increase of QH relative content ratio from 1:1.5 (Film 2), 1:1 (Film 1) to 1.5:1 (Film 4). It was due to that QH have many branch chains on its backbone, which resulted in the roughness of the blended films increased. When the ratio of QH/CMC reached up to 2:1, the roughness of blend Film 3 had a little decrease compared with Film 4, but still higher than that of Film 1 and Film 2.

### 3.4. Mechanical Properties

**Figure 7:**
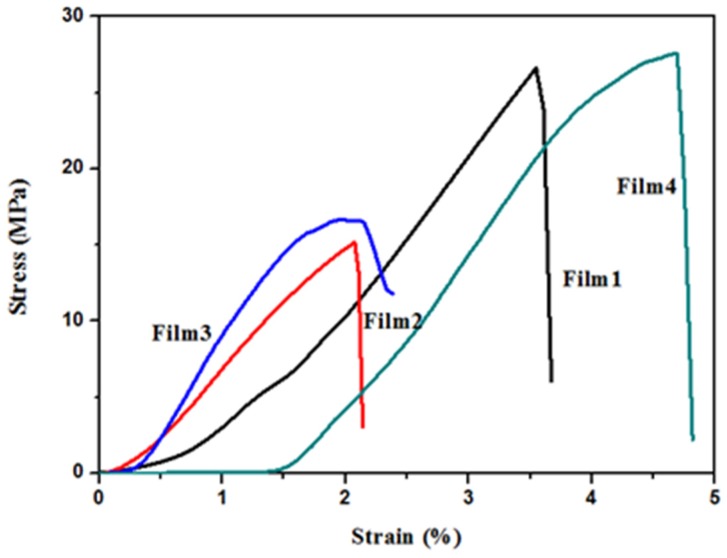
The tensile stress-strain curves of Film 1, Film 2, Film 3, and Film 4.

The tensile stress, tensile strain at break, and Young’s modulus of films are shown in Table 3, and the tensile stress-strain curves of the blended films are shown in Figure 7. As can been seen, it was found that the tensile strength of blended films increased with the increase of QH/CMC content ratio from 1:1.5 (Film 2), 1:1 (Film 1) to 1.5:1 (Film 4), and the maximum value 27.4 MPa was obtained in Film 4; nevertheless, the tensile strength of Film 3 (QH/CMC, 2:1) reduced obviously when the QH relative content further increased. It was mainly caused by the intermolecular hydrogen bonding and electrostatic interaction between QH and CMC. That is to say, the miscibility of the films increases with the QH/CMC content in the appropriate range. From Film 2 to Film 1 to Film 4, the quantity and intensity of the hydrogen bonding and electrostatic interaction increased with the QH content increasing. Therefore, the tensile strength of the films increased consequently. However, with the relative content of QH further increasing, the miscibility of the films decreased, which resulted in the hydrogen bonding and electrostatic force were not formed enough between QH and CMC because of the relatively small amount of CMC. That is to say, the formed interaction force was too weak to support the structure of the Film 3, resulting in the sharp decrease of tensile strength of Film 3. The results discussed above indicated that the mechanical properties of the blended films were greatly affected by the QH content, and the excess QH would reduce the mechanical property of the blended films.

**Table 3:** Tensile testing results of the four blended films.

Sample	Tensile Strength (MPa)	Tensile Strain at Break (%)	Young‘s Modulus (MPa)
Film 1	25.2 ± 2.3	2.8 ± 0.7	1319.9 ± 102.7
Film 2	14.2 ± 0.9	2.6 ± 0.9	842.0 ± 11.1
Film 3	14.8 ± 1.6	1.7 ± 0.5	1177.2 ± 126.5
Film 4	27.0 ± 1.5	3.9 ± 0.8	1118.5 ± 53.5

### 3.5. UV-Vis Transparency of Films

**Figure 8:**
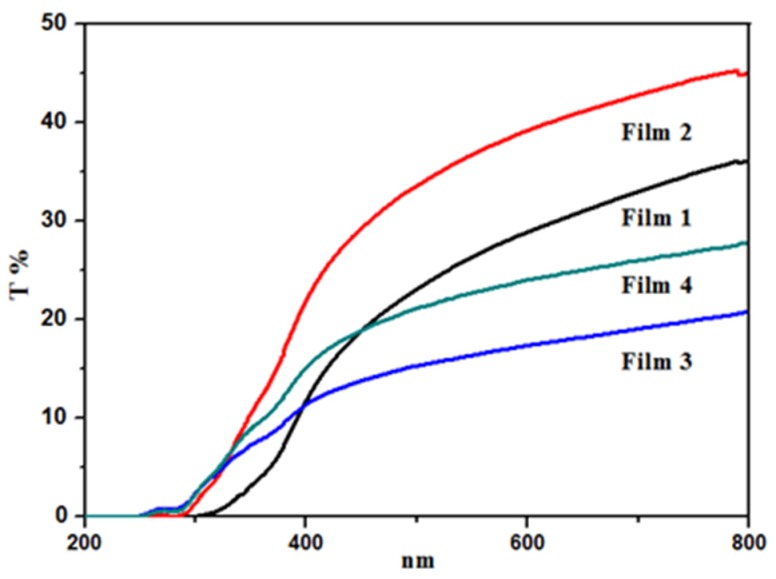
UV-Vis spectra of blend Film 1, Film 2, Film 3, and Film 4.

Generally, transparency of film is an assistant criterion to judge the miscibility of blended films. The light transmittances of the blended films at wavelength of 200–800 nm are shown in Figure 8. It can be seen obviously that the transmittance curve of each film rose gradually with the increase of wavelength. Comparing the four blended films, the transparencies of the blended films increased with the QH/CMC content ratio decreasing in visible light spectrum, and it had the following order: Film 2 > Film 1 > Film 4 > Film 3. In other words, a relatively high content of CMC in the blended films is beneficial for the transparency of the blended films.

### 3.6. Water Vapor Permeability (WVP)

**Figure 9:**
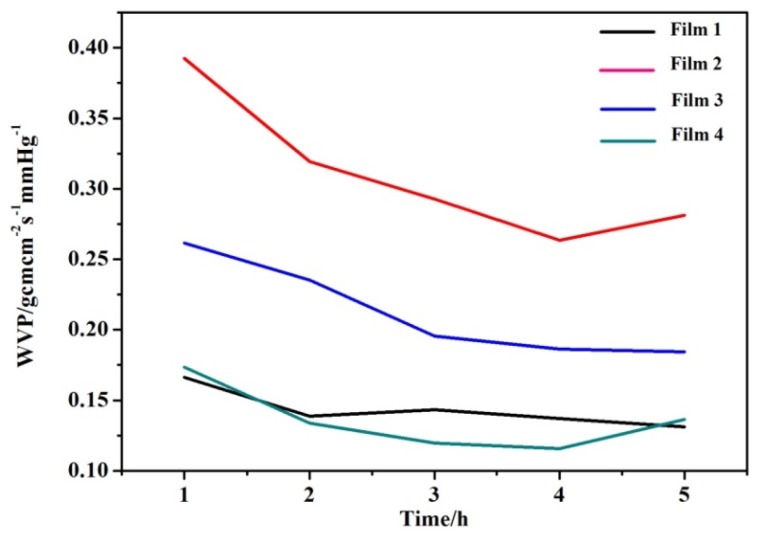
The water vapor permeability of blend Film 1, Film 2, Film 3, and Film 4.

The WVP values of the blended films are important measures for the applications of packaging materials. One of the main functions of food packaging is to avoid or minimize moisture transfer between food and the surrounding atmosphere. Low WVP widens the application of the composite packaging film, especially in a highly humid environment. The WVP curves of the four blended films are shown in Figure 9. It was found that all the four blended films showed a relatively lower WVP, suggesting that the fabricated films have the capability to withstand water vapor. The WVP values of blended films had the following order: Film 2 > Film 3 > Film 1 > Film 4, indicating that the WVP values decreased with the increase of QH/CMC content ratio from 1:1.5 (Film 2), 1:1 (Film 1) to 1.5:1 (Film 4). However, an interesting result was observed. When the QH/CMC content ratio was further increased to 2:1 (Film 3), the WVP value of Film 3 increased instead. The causes of this phenomenon are the different densification of the four blended films. As discussed in the analysis of the blended films’ mechanical properties, the quantity and intensity of the hydrogen bonding and electrostatic force increased with the QH/CMC content ratio increasing, which led to the tightness of internal structure of blended films. It also prevented water molecules from diffusing through the films. Therefore, the WVP of films decreased with QH/CMC content ratio increasing. When the QH/CMC content ratio was further increased to 2:1 (Film 3), the hydrogen bonding and electrostatic interaction were not formed enough between QH and CMC because of the relatively small amount of CMC. Then, the structure of blend Film 3 became loose again and the WVP of Film 3 increased. Therefore, the interactions between components have obvious influence on the densification of the blended films [37]. The results indicated that the WVP of the blended films is related to the intensity of interaction force between QH and CMC, and it is in accordance with the tensile strength of the blended films.

## 4. Conclusions

A series of blended films of QH and CMC were successfully fabricated in this study, and they were linked by the hydrogen bonding and electrostatic interaction. The blended films with different proportions of QH and CMC were also studied to determine their properties. From the results of mechanical properties and water vapor permeability, it was found that the intensity of hydrogen bonding and electrostatic interaction between QH and CMC increased with the increasin QH/CMC content ratio in the appropriate range, which made the film structurally dense, resulting in the tensile strength increase and WVP film decrease, respectively. However, hydrogen bonding and electrostatic interaction were not formed enough to support the structure of the film when excess QH was added, which led to the film tensile strength decrease and the WVP increase. The light transmittances of the blended films increased with the decrease of the QH/CMC content ratio. The prepared films show good strength, low WVP, and good light transmittances, which are beneficial for the applications of films as packaging materials. Besides, compared with the petroleum-based plastics, the films fabricated from QH and CMC have biocompatible, nontoxic, and biodegradable properties. Therefore, the films fabricated by blending CMC with QH might become attractive in the application of packaging materials to replace the traditional petroleum-based packaging materials.
